# *Porphyromonas gingivalis* HmuY stimulates expression of Bcl-2 and Fas by human CD3^+^ T cells

**DOI:** 10.1186/1471-2180-13-206

**Published:** 2013-09-11

**Authors:** Paulo C Carvalho-Filho, Soraya C Trindade, Teresa Olczak, Geraldo P Sampaio, Milton G Oliveira-Neto, Heidiane A Santos, Bianca F P Pereira, Lilia Moura-Costa, Márcia T Xavier, Roberto Meyer

**Affiliations:** 1 Department of Immunology, Federal University of Bahia, Salvador, BA, Brazil; 2 Department of Periodontics, Feira de Santana State University, Salvador, BA, Brazil; 3 Department of Biotechnology, University of Wroclaw, Wroclaw, Poland; 4 Department of Dentistry, Bahiana School of Medicine and Public Health, Salvador, BA, Brazil; 5 LABIMUNO-ICS-UFBA, Av. Reitor Miguel Calmon, s/n; Vale do Canela, Salvador, BA 40110100, Brazil

**Keywords:** *Porphyromonas gingivalis*, HmuY, Bcl-2, Fas, Apoptosis

## Abstract

**Background:**

Apoptosis is a highly controlled process of cell death that can be induced by periodontopathogens. The present study aimed to investigate the expression of Fas and Bcl-2 proteins by CD3^+^ T cells *in vitro* under stimulation by total *Porphyromonas gingivalis* antigens and purified recombinant *P. gingivalis* HmuY protein.

**Results:**

CD3^+^ T cells derived from CP patients and stimulated with HmuY expressed higher levels of Bcl-2 compared to identical cells stimulated with *P. gingivalis* crude extract or cells derived from NP control subjects (*p* = 0.043).

**Conclusion:**

The authors hypothesize that *P. gingivalis* HmuY plays a role in the pathogenesis of chronic periodontitis, possibly by reducing or delaying apoptosis in T cells through a pathway involving the Bcl-2 protein.

## Background

*Porphyromonas gingivalis* is one of the most important etiologic agents involved in chronic periodontitis (CP), an infectious and multifactorial disease that leads to the destruction of the periodontium. During the infective process, bacteria acquire nutrients to survive and multiply at the site of infection. Heme, one of these nutrients, is an iron-dependent cofactor of many indispensable enzymes and proteins. *P. gingivalis* acquires heme from host heme-binding proteins through proteolysis and transports heme into the bacterial cell using outer membrane receptors [1]. A previously characterized heme uptake system in *P. gingivalis* utilizes two proteins: HmuY, which scavenges heme from host hemoproteins, and HmuR [2-4], which transports the nutrient across bacterial cell membranes. These proteins are virulent factors, yet they can be antigenic and immunogenic as well, potentially affecting a host’s immune system with respect to stability and resistance.

HmuY is a membrane-associated lipoprotein identified in *P. gingivalis* 381, W50, W83, and ATCC 33277 strains [2,3]. Homologous amino acid sequences have also been identified in *Bacteroides fragilis* and *B. thetaiotaomicron*[3]. In *P. gingivalis* strains, the *hmuY* gene is located in an operon with a *hmuR* gene and four other uncharacterized genes [2,3]. HmuY is exposed on the cell surface and attached to the outer membrane, or is released into vesicles in a soluble form [4,5]. This protein is produced constitutively at low levels in bacterial cultures grown under high-iron/heme conditions, and also at higher levels in bacteria growing under low-iron/heme conditions, such as those typically found in dental plaque [3,5]. HmuY may play a role not only in heme acquisition, but also in biofilm accumulation on abiotic surfaces [5]. Furthermore, it has been suggested that HmuY, a surface-exposed protein, might be recognized during the immune response occurring in chronic periodontitis. In addition, recent studies have demonstrated that anti-HmuY antibodies, whose production is increased in CP patients [6], can inhibit *in vitro* biofilm formation [5].

Inflammatory sites resulting from periodontal disease contain plasma cells, T and B lymphocytes and macrophages [7]. Periodontal lesions are characterized by a persistence of infiltrating inflammatory cells, which may be responsible for the chronic disease. Recently, the presence of regulatory T cells (Treg) [8,9] and Th17 cells [10,11] has been demonstrated in periodontal tissues, thus highlighting their role in the immunoregulation of periodontal disease. The clinical implications of recent studies can be evidenced by the identified genetic expression of cytokines Th1/Th2 and Treg/Th17 in peripheral blood, as well as in salivary transcriptomes that are currently undergoing testing as potential markers of disease susceptibility [12]. CD4^+^ and CD8^+^ T cells are present in periodontal lesions and may be activated towards memory lymphocytes. This cellular subset stimulates the production of proinflammatory cytokines that induce bone resorption by way of an imbalance in the RANK-RANKL-OPG axis, thereby promoting the differentiation of pre-osteoclasts into mature/activated osteoclasts [13].

At sites of chronic inflammation, apoptosis associated with cell destruction occurs in human gingival cells stimulated by bacterial infection, which is also important for mucosal membrane homeostasis [14]. The main pro-apoptotic protein is Fas, APO-1/Fas (CD95/TNFRSF6), a member of the tumor necrosis factor (TNF) or nerve factor receptors superfamily [15]. The APO-1/Fas receptor plays a central role in the physiological regulation of programmed cell death (apoptosis). Bcl-2 is a member of the family of anti-apoptotic proteins that prevent or delay cell death induced by a variety of stimuli [16,17]. The mitochondrial Bcl-2 family is comprised of several anti-apoptotic members, including Bcl-2, Bcl-XL, Bcl-w and Mcl-1, in addition to many pro-apoptotic members, such as Bax, Bak, Bok, Bid, Bad, Puma, Bmf, Bim, Bok, Noxa and Hrk/DP5 [17].

The modulation of cell-mediated immunity by microorganisms has been demonstrated in periodontal disease since the 1970s [18-21]. By contrast, programmed cell death, as well as the expression of proteins Fas and Bcl-2 in peripheral blood mononuclear cells (PBMC) under stimulation by periodontopathogens, have not received appropriate consideration. To investigate the hypothesis that *P. gingivalis* antigens, including HmuY, may be involved in the apoptotic response of T cells, the present study aimed to evaluate the expression of Fas and Bcl-2 under stimulation by total *P. gingivalis* antigens present in sonicated crude extract, as well as by purified recombinant *P. gingivalis* HmuY protein.

## Results

The periodontitis patients and the healthy subjects were comparable regarding to the gender, age and number of teeth present in the mouth as shown in the Table 1. As expected, periodontal condition were worse in the periodontitis patients.

**Table 1:** Clinical findings of control subjects without periodontitis (NP) and patients with chronic periodontitis (CP)

	**NP**	**CP**	***P***
Number of Men /Women	3/18	5/13	0.622
Age (years) (Mean ± SD)	36 ±15.67	40.11 ± 14.67	0.231
Number of Teeth (Mean ± SD)	22.56 ±7.45	22.65 ± 7.12	0.914
% BOP (Mean ± SD)	6.31 ± 13.93	35.82 ± 26.28	0.001
% PD ≥ 4 (Mean ± SD)	1.31 ± 1.94	14.71 ± 10.52	0.001
% CAL ≥ 3 (Mean ± SD)	12.26 ± 18.96	28.79 ± 26.04	0.059

*SD* Standard Deviation, *BOP* Bleeding on Probing, *PD* Probing Depth, *CAL* Clinical Attachment Loss.

**Figure 1:**
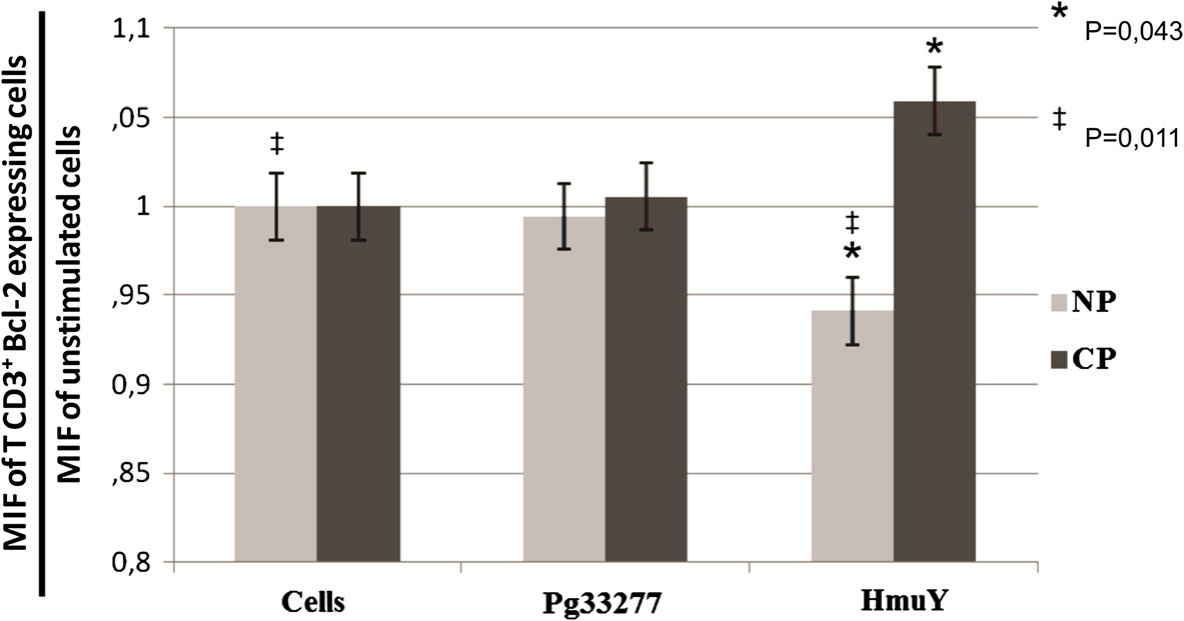
**Bcl-2 expression by CD3**^**+ **^**T cells derived from chronic periodontitis (CP) patients and subjects without periodontitis (NP) upon stimulation (48 h) with *P. gingivalis *ATCC 33277 crude extract (Pg33277), purified recombinant *P. gingivalis *HmuY protein (HmuY), or without stimulus (Cells) as evaluated by flow cytometry.** *p = 0.043, ^‡^p = 0,011.

The data presented herein refer to CD3^+^ T cells and demonstrate that higher levels of HmuY-induced Bcl-2 expression were obtained in cells derived from CP subjects in comparison to individuals without periodontal disease (NP) (*P* = 0.043) (Figure 1). On the other hand, it was observed statistically significant lower levels of Bcl-2 expression in cells derived from NP subjects stimulated with HmuY in comparison with the cells derived from the same group cultured only with culture medium (*P* = 0,011). Furthermore, the cells from CP patients exhibited a tendency towards increased Bcl-2 expression under stimulation by HmuY when compared to those stimulated by *P. gingivalis* crude extract or to cells cultured in the absence of stimulus (Figure 1).

**Figure 2:**
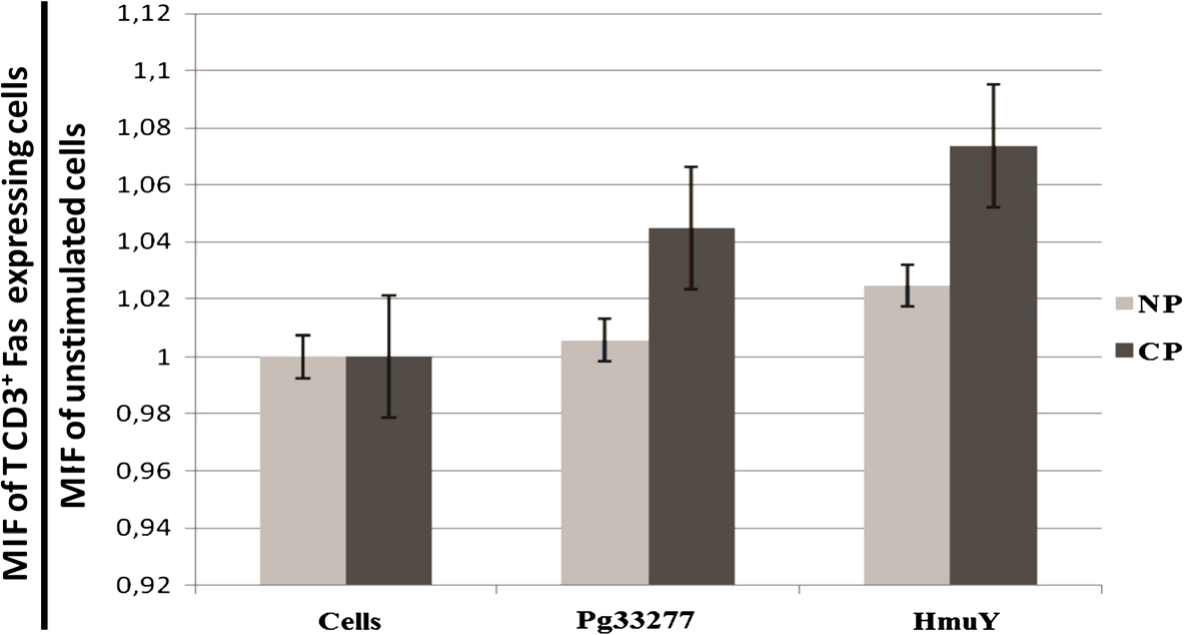
**Fas expression by CD3**^**+ **^**T cells derived from chronic periodontitis (CP) patients and subjects without periodontitis (NP) upon stimulation (48 h) with *P. gingivalis *ATCC 33277 crude extract (Pg33277), purified recombinant *P. gingivalis *HmuY protein (HmuY), or without stimulus (Cells) as evaluated by flow cytometry.**

Under HmuY stimulation, no statistically significant differences in Fas expression were observed between the two groups studied. However, a tendency toward elevated levels of Fas expression were observed in CD3^+^ T cells derived from CP patients when compared to NP subjects (Figure 2).

## Discussion

This study demonstrated that HmuY was able to stimulate higher expression of Bcl-2 by T CD3^+^ cells derived from CP patients after 48 h, suggesting that this molecule induces an increase in cell survival by inhibiting apoptosis. Elevated expression levels of Bcl-2 can prevent cellular apoptosis, thereby inducing inflammatory cells to remain locally in the periodontal tissue, causing consequent excessive cytokine secretion which leads to the progressive destruction of periodontal tissues. Apoptosis is a form of cell death mediated by caspases with specific morphological and anti-inflammatory features [22]. In the absence of phagocytosis, apoptotic bodies may undergo lysis and secondary necrosis, also known as late apoptosis, releasing necrotic cell content including molecules that act as promoters of inflammatory response [23]. Conversely, the uptake of apoptotic bodies suppresses the secretion of inflammatory mediators in activated macrophages [24]. In chronic periodontitis, the infiltrating cells in periodontal lesions are stimulated with a variety of bacterial antigens. Therefore, it is possible that the continuous stimulation of host cells would enhance the possibility of apoptosis activation in lymphocytes.

Recent data [25] has shown that *P. gingivalis* total antigens, as well as purified recombinant *P. gingivalis* HmuY, stimulate late apoptosis in PBMCs derived from CP patients. This finding suggests that although the protein is capable of signaling the apoptotic pathway, the stimulated cell is unable to terminate the apoptotic process that leads to cell death, thereby secondary necrosis is the resulting mechanism.

The present findings corroborate another study [26] that found a higher number of Bcl-2 positive cells in the inflammatory infiltrate of periodontitis patients, suggesting that the Bcl-2 protein may play a role in the control of apoptosis in inflammatory cells. The up-regulation of Bcl-2 was observed in epithelial cells in response to *Porphyromonas gingivalis* gingipains, [27,28] which indicates that this bacteria can survive in the cellular environment by evading the host immune response.

The present study also found decreased Bcl-2 expression in CD3^+^ T cells derived from subjects without periodontitis upon HmuY stimulation. These findings with respect to NP individuals suggest a lack of prior immune stimulation by *P. gingivalis* antigens in comparison to CP patients, whose immune systems are constitutively primed by bacterial antigens at sites of periodontal lesions.

Another interesting result was that cells from CP patients exhibited increased Bcl-2 expression under stimulation by HmuY when compared to those stimulated by *P. gingivalis* crude extract or to cells cultured in the absence of stimulus. However, these differences were not statistically significant. Other investigations have been done to confirm or refute these preliminary findings. It’s important to emphasize that the concentrations employed for each antigen was previously tested [6,25,29]. In this study, it was used 2.5 μg/mL of HmuY versus 0.5 μg/mL of crude extract (5fold more of the recombinant protein). The capacity of only one molecule to induce a immune response is very low in comparison to a crude extract, which contains diverse somatic proteins and thus, can exposure many different epitopes to be recognized.

Fas and Fas ligand are expressed in inflamed gingival tissue, as well as in the lymphocytes that accumulate in chronic periodontal lesions. The Fas-positive lymphocytes isolated from these lesions induce apoptosis by the anti-Fas antibody, which mimics the function of Fas ligand, while peripheral lymphocytes resist apoptosis under stimulation with this same antibody [30]. Thus, it has been suggested that the absence of Fas-mediated apoptosis in activated lymphocytes could contribute to chronic disease and that exogenous Fas ligand may be a candidate for protection against the profile of chronic disease. In the present study, slightly elevated Fas expression by CD3^+^ T lymphocytes stimulated with *P. gingivalis* total antigens and HmuY was observed. The authors hypothesize that the lack of statistical significance in the results presented herein indicates that this may not be the primary pathway that is being stimulated. Nonetheless, it is possible that the relatively small sample size employed herein was unable to produce demonstrable results with respect to Fas expression under the established experimental conditions.

In addition, the present study showed that HmuY may also be an important stimulus used by *P. gingivalis* to induce increased expression of Bcl-2 in CD3^+^ T cells derived from CP patients. An inflammatory outcome is the most expected one following contact between host cells and *P. gingivalis* antigens, including HmuY, due to the association with necrotic cell death and membrane disruption, in addition to the exhibition of pro-inflammatory moieties. The absence or delay of apoptosis may play an important role in survival of PBMCs in CP patients and may even contribute to the chronicity of this disease. Further studies should be conducted to evaluate the receptor responding to the HmuY protein and identify the pathway involved in programmed cell death, as well as the role of HmuY in *P. gingivalis* infection *in vivo*.

The *P. gingivalis* HmuY recombinant protein was also observed to inhibit Bcl-2 expression in PBMCs obtained from NP individuals, which was not the case in cells taken from CP patients. This protein is known to play an important in the “mounting” of host immune response by preventing apoptosis in lymphocytes. However, an exacerbated immune response against a bacterial challenge [1,3-5] is known to be a factor that is crucial to the pathogenesis of periodontitis. Accordingly, the inhibition of Bcl-2 in individuals without periodontitis may be one of the underlying mechanisms that prevent these individuals from developing the disease. A recent report that evaluated individuals with similar clinical characteristics [25] revealed that HmuY induced delayed apoptosis, as evidenced by the fact that cultivated cells stimulated with this recombinant protein presented concomitant labeling with annexin V and propidium iodide.

## Conclusions

Decreased Bcl-2 expression in CD3^+^ T cells was also shown to be a preliminary indicator of a mechanism that may be capable of preventing some individuals from developing CP, i.e., the cells that undergo apoptosis do not consequently produce elevated levels of proinflammatory mediators, which are responsible for tissue degradation. The absence or delay in the apoptosis process may play an important role in the survival of PBMCs in CP patients in addition to possibly prolonging the chronic form of this disease.

## Methods

A total of 18 patients with CP and 21 control subjects without periodontitis (NP) were recruited between 2009 and 2010 at the Municipal Specialized Dentistry Center (Salvador, Bahia) and from the College of Dentistry at the Federal University of Bahia. The following exclusion criteria were established: presence of diabetes, cardiovascular disease, pregnancy, auto-immune disease, tobacco use, prior periodontal treatment, use of anti-inflammatory drugs within two months prior to inclusion and/or antibiotic drug use less than six months before inclusion. Informed written consent was obtained from all study subjects in accordance with guidelines established by the Brazilian Health Council. The present study was approved by the Institutional Review Board of the Climério de Oliveira Maternity Hospital (Protocol no. 053/2010).

Periodontal examination was performed by a single, previously calibrated examiner (P.C.C.F.) (kappa inter-examiner agreement value = 0.932) using a Williams periodontal probe (Hu Friedy, Chicago, IL, USA). Investigated criteria included bleeding on probing (BOP), clinical attachment level (CAL) and probing depth (PD) at six sites for each tooth. Patients met the established criteria for periodontitis when the following conditions were satisfied: four or more teeth with one or more sites presenting probing depths ≥ 4 mm with a clinical attachment loss ≥ 3 mm and bleeding on probing present at the same site [31]. The chronic character of disease was evaluated in accordance with guidelines established by the American Academy of Periodontology [32].

Crude extract from *P. gingivalis* ATCC 33277 wild-type strain was obtained as previously described [33] and prepared for use at a final concentration of 0.5 μg/mL. The *P. gingivalis* HmuY polypeptide lacking the first 25 residues (NCBI accession no. CAM 31898) was overexpressed using pHmuY11 plasmid and *Escherichia coli* ER2566 cells (New England Biolabs, MA, USA), then purified from a soluble fraction of *E. coli* lysate as previously described [5]. Contaminating endotoxins were removed from the HmuY sample using Detoxi-Gel Endotoxin Removing Columns (Thermo Scientific, Rockford, IL, USA). HmuY was prepared at a final concentration of 2.5 μg/mL.

Twenty milliliters of peripheral venous blood were drawn from each individual and collected in heparin tubes. Mononuclear cells (PBMC) were obtained from peripheral blood samples and purified by density centrifugation in accordance with manufacturer guidelines (SepCell, StemCell Technologies Inc., USA). All cells were washed twice in RPMI (Roswell Park Memorial Institute) medium (LGCBio, São Paulo, SP, Brazil) and PBMCs were cultured in flat-bottom 24-well plates (10^6^ cells/well) in RPMI medium containing 10% fetal calf serum (complement proteins inactivated by heat) and 1% antibiotic/antimycotic solution (R&D Systems, Minneapolis, MN, USA). All cultures were grown for 48 h at 37°C under 5% CO_2_ in humid conditions. Cells were also incubated with 5 μg/mL of pokeweed mitogen (PWM) as a positive control, 0.5 μg/mL of *P. gingivalis* extract (ATCC 33277), 2.5 μg/mL of HmuY, or in the absence of antigens (Cells).

All PBMCs were collected by centrifugation and resuspended in 500 μL of 1×binding buffer, then incubated with fluorescently labeled antibodies in accordance with manufacturer instructions (Life Science, Carlsbad, CA, USA). To identify the expression of the anti-apoptotic protein Bcl-2 and the Fas death receptor, mouse anti-human Bcl-2 (IgG1 kappa) conjugated with PE CY, mouse anti-human CD95 (IgG1) conjugated with fluorescein isothiocyanate (FITC), mouse anti-human CD3 conjugated with PerCP CY (IgG2a), or isotype-matched controls antibodies were used. The triple expression of CD3, CD4 and CD8 was identified by flow cytometry using the FITC, PE CY and PerCP CY signal detectors and BD FACSCalibur equipment (BD Facscalibur, Franklin Lakes, NJ, USA).

Clinical variables were described in terms of means±standard deviations (mean±SD). Student’s *t*-test was used to compare clinical features among groups. The Mann–Whitney test was used to assess differences among groups with respect to immunological data in the absence of normal distribution. Statistical significance was considered when *p* < 0.05. SPSS 17.0 (Statistical Package for Social Science, USA) software was used to perform all statistical analyses.

## Competing interests

The authors have declared no competing of interests.

## Authors’ contributions

PCCF, SCT and MTX were responsible for the study design. PCCF, SCT and MTX analyzed and interpreted the data. PCCF, SCT and MTX wrote the report. PCCF, GPS, MGON, HAS, BFPP did the laboratory work. RM, LMC and TO helped to draft the manuscript. All authors read, commented and approved the final article.
